# Value-added uses for crude glycerol--a byproduct of biodiesel production

**DOI:** 10.1186/1754-6834-5-13

**Published:** 2012-03-14

**Authors:** Fangxia Yang, Milford A Hanna, Runcang Sun

**Affiliations:** 1 College of forestry, Northwest Agricultural and Forestry University, Yangling 712100, P. R. China; 2 Department of Biological systems Engineering, Industrial Agricultural Products Center, University of Nebraska-Lincoln, Lincoln, NE 68583, USA; 3 Institute of Biomass Chemistry and Technology, Beijing Forestry University, Beijing 100083, China

**Keywords:** Crude glycerol, Value-added utilization

## Abstract

Biodiesel is a promising alternative, and renewable, fuel. As its production increases, so does production of the principle co-product, crude glycerol. The effective utilization of crude glycerol will contribute to the viability of biodiesel. In this review, composition and quality factors of crude glycerol are discussed. The value-added utilization opportunities of crude glycerol are reviewed. The majority of crude glycerol is used as feedstock for production of other value-added chemicals, followed by animal feeds.

## Background

The dramatic increase in demand for transportation fuels and the increase in environmental concerns, coupled with diminishing crude oil reserves, have increased the emphasis on renewable energy. Biodiesel, one of the promising alternative and renewable fuels, has been viewed with increasing interest and its production capacity has been well developed in recent years. Although world biodiesel production was expected to reach a high capacity, in fact, it is less than the anticipated target and has increased at a slower rate [1]. The main reason is its relatively high production cost. Utilization of the glycerol co-product is one of the promising options for lowering the production cost.

Biodiesel production will generate about 10% (w/w) glycerol as the main byproduct. In other words, every gallon of biodiesel produced generates approximately 1.05 pounds of glycerol. This indicates a 30-million-gallon-per-year plant will generate about 11,500 tonnes of 99.9 percent pure glycerin. It was projected that the world biodiesel market would reach 37 billion gallons by 2016, which implied that approximately 4 billion gallons of crude glycerol would be produced [2]. Too much surplus of crude glycerol from biodiesel production will impact the refined glycerol market. For example, in 2007, the refined glycerol's price was painfully low, approximately $0.30 per pound (compared to $0.70 before the expansion of biodiesel production) in the United States. Accordingly, the price of crude glycerol decreased from about $0.25 per pound to $0.05 per pound [3]. Therefore, development of sustainable processes for utilizing this organic raw material is imperative.

Since purified glycerol is a high-value and commercial chemical with thousands of uses, the crude glycerol presents great opportunities for new applications. For that reason, more attention is being paid to the utilization of crude glycerol from biodiesel production in order to defray the production cost of biodiesel and to promote biodiesel industrialization on a large scale. Although intensive investigations have focused on utilizing crude glycerol directly, review papers on crude glycerol utilization are scarce. This review mainly addresses the current and potential value-added applications of crude glycerol from biodiesel production.

## Chemical compositions of crude glycerol

The chemical composition of crude glycerol mainly varies with the type of catalyst used to produce biodiesel, the transesterification efficiency, recovery efficiency of the biodiesel, other impurities in the feedstock, and whether the methanol and catalysts were recovered. All of these considerations contribute to the composition of the crude glycerol fraction. For instance, Hansen *et al*. [4] studied the chemical compositions of 11 crude glycerol collected from 7 Australian biodiesel producers and indicated that the glycerol content ranged between 38% and 96%, with some samples including more than 14% methanol and 29% ash. Such variations would be expected with small conversion facilities. In most cases, biodiesel production involves the use of methanol and a homogeneous alkaline catalyst, such as sodium methoxide and potassium hydroxide. Accordingly, methanol, soap, catalysts, salts, non-glycerol organic matter, and water impurities usually are contained in the crude glycerol. For example, crude glycerol from sunflower oil biodiesel production had the following composition (w/w): 30% glycerol, 50% methanol, 13% soap, 2% moisture, approximately 2-3% salts (primarily sodium and potassium), and 2-3% other impurities [5]. Moreover, while the same feedstocks were employed, the crude glycerol from alkali- and lipase-catalyzed transesterifications contained different purities of glycerol [6]. The salt content in crude glycerol, from biodiesel production via homogeneous alkaline catalysts, ranged from 5% to 7% [7] which makes the conventional purification techniques more costly. Heterogeneous processes using enzymes and solid metal-oxide catalysts have been promoted as good alternatives to homogeneous alkaline catalysts in terms of improving the quality of crude glycerol. However, even in heterogeneous transesterification processes, impurities existing in the natural raw feedstocks tend to accumulate in the glycerol phase. Therefore, purification of crude glycerol is required, in most cases, to remove impurities in order to meet the requirements of existing and emerging uses.

## Value-added opportunities for crude glycerol

Worldwide, crude glycerol derived from biodiesel conversion has increased from 200, 000 tonnes in 2004 [8] to 1.224 million tonnes in 2008 [1]. Meanwhile, the global market for refined glycerol was estimated to be roughly 900, 000 tonnes in 2005 [9]. Therefore, it is of great importance for scientists to find new applications for refined and crude glycerol. Recently, numerous papers have been published on direct utilization of crude glycerol from biodiesel production. In the following sections, detailed discussions on utilization of crude glycerol are presented.

### Animal feedstuff

Using glycerol as a feed ingredient for animals dates back to the 1970s [10]. However, glycerol's utilization in feeds has been limited by the availability of glycerol [3]. Recently, the possibilities of using crude glycerol from biodiesel in feeds have been investigated because of the increase in the price of corn and the surplus of crude glycerol.

#### Crude glycerol in non-ruminant diets

Glycerol has high absorption rates and is good energy source. Once absorbed, it can be converted to glucose for energy production in the liver of animals by the enzyme glycerol kinase [3]. Crude glycerol samples, from different biodiesel producers, were analyzed as energy sources. The digestible energy (DE) values for 85% of the crude glycerol samples were in the range of 14.9-15.3 MJ/kg with metabolizable energy (ME) values in the range of 13.9-14.7 MJ/kg [11]. Crude glycerol was an excellent source of calories for non-ruminants, for example, the ME determined in broilers, laying hens and swine were 15.2, 15.9 and 13.4 MJ ME/kg, respectively [3]. In growing pigs and laying hens, 14.0 MJ/kg apparent DE [12] and 15.9 MJ/kg nitrogen-corrected apparent ME (AMEn) [13] were reported, respectively, which implied that crude glycerol was used efficiently. The AMEn of crude glycerol was metabolized efficiently by broiler chickens with an AMEn of 14.4 MJ/kg. That was very similar to the general energy (GE) of 15.2 MJ/kg [14]. In nursery pigs, the GE concentration of crude glycerol depended on the concentration of glycerol, methanol, and fatty acids, with ME as a percent of GE averaging 85.4% [15].

Although crude glycerol can be added to animal feed, excess glycerol in the animal diet may affect normal physiological metabolism. A few manuscripts have been published that focused on the levels of crude glycerol fed and the performance of crude glycerol in animal feeds. The improvement of daily gains by pigs depended on the actual intake of glycerol during the growing period but not on the finishing period. The dietary treatments had no significant effects on meat quality [16,17]. When crude glycerol was added to the diets of weaned pigs, at levels up to 10%, the feed performance was enhanced [18]. Up to 9% crude glycerol could be added to the diets of lactating sows with performances similar to sows fed standard corn-soybean meal control diets [19]. No detrimental effects, with respect to egg performance, egg quality, nutrient retention, and metabolizable energy, were found when crude glycerol was incorporated at a level of 6% in the diet of laying hens [20]. In broiler diets, increasing the intake level of crude glycerol increased feed conversion ratio but did not affect growth performance and nutrient digestibility [21]. Crude glycerol was used effectively at levels of 2.5 or 5%. But the use of 10% crude glycerol resulted in poor feed flow. The influences of levels and quality of crude glycerol on pellet quality needs further study [22].

#### Crude glycerol in ruminant diets

Crude glycerol, added at up to 15% dry matter in the diets of finishing lambs, could improve feedlot performance, especially during the first 14 d, and had no associated effect on carcass characteristics [23]. Compared to medium-quality hay, diets for meat goats with up to 5% crude glycerol proved to be beneficial [24]. In addition, the inclusion of purified glycerol at up to 15% of the dry matter ration of lactating dairy cows was possible, without deleterious effects on feed intake, milk production, and yield [25,26]. When crude glycerol was added at levels of 8% or less, based on dry matter in cattle finishing diets, it improved weight gain and feed efficiency [27]. Apart from the above mentioned investigations, a patent described approaches for using or incorporating crude glycerol into animal feeds as well as feeding recommendations [28].

In all, the use of crude glycerol as an animal feed component has great potential for replacing corn in diets, and is gaining increasing attention. However, one must be aware of the presence of potential hazardous impurities in crude glycerol from biodiesel. For example, residual levels of potassium may result in wet litter or imbalances in dietary electrolyte balance in broilers [22]. The levels of methanol must be minimized because of its toxicity [3,12,22,26]. More attention should be paid to the crude glycerol from small scale biodiesel facilities that use simple batch distillation or evaporation techniques.

### Feedstocks for chemicals

#### Chemicals produced via biological conversions

##### 1, 3-propanediol

The anaerobic fermentative production 1, 3-propanediol is the most promising option for biological conversion of glycerol. Mu *et al*. [6] demonstrated that crude glycerol could be used directly for the production of 1, 3-propanediol in fed-batch cultures of *Klebsiella pneumoniae*. The differences between the final 1, 3-propanediol concentrations were small for crude glycerol from the methanolysis of soybean oil by alkali- (51.3 g/L) and lipase-catalysis (53 g/L). This implied that the composition of crude glycerol had little effect on the biological conversion and a low fermentation cost could be expected. Further, the production of 1,3-propanediol by *K.pneumoniae *was optimized using response surface methodology. The maximum yield of 1, 3-propanediol was 13.8 g/L [29]. More recently, the production of 1, 3-propanediol, from crude glycerol from Jatropha biodiesel by *K.pneumoniae *ATCC 15380, was optimized. The obtained 1, 3-propanediol yield, purity and recovery were 56 g/L, 99.7% and 34%, respectively [30]. Additionally, an incorporated bioprocess that combined biodiesel production by lipase with microbial production of 1, 3-propanediol by *K.pneumoniae *was developed in a hollow fiber membrane. The bioprocess avoided glycerol inhibition on lipase and reduced the production cost [31].

*Clostridium butyricum *also could be used to produce 1, 3-propanediol from crude glycerol. For example, *C. butyricum *VPI 3266 was able to produce 1, 3-propanediol from crude glycerol on a synthetic medium. Trivial differences were found between commercial glycerol and crude glycerol [32]. *C.butyricum *strain F2b and *C.butyricum *VPI 1718 potentially could convert crude glycerol to 1, 3-propanediol [33-35]. In order to avoid the isolation of 1,3-propanediol from crude glycerol fermentation media, a one vessel bio- and chemo-catalytic process was developed to convert crude glycerol to secondary amines directly in a biphasic system without intermediate separation of 1, 3-propanediol [36]. Additionally, Chatzifragkou *et al*. [37] studied the effects of different impurities in crude glycerol on 1, 3-propanediol production by *C.butyricum*. The double bond from long-chain fatty acids or methyl esters might influence the growth performance of the microorganism, methanol did not affect the microbial conversion and the presence of NaCl had certain effect during a continuous process but not a batch process.

##### Citric acid

A few reports are available on the use of crude glycerol for citric acid biosynthesis. The production of citric acid from crude glycerol by *Yarrowia lipolytica *ACA-DC 50109 was not only similar to that obtained from sugar-based conventional media [33] but also single-cell oil and citric acid were produced simultaneously [34,38]. When a fed-batch fermentation by acetate-negative mutants of *Y. lipolytica *Wratislavia AWG7 strain was used to ferment crude glycerol, the final concentration of citric acid was 131.5 g/L, similar to that obtained from pure glycerol (139 g/L). On the other hand, when *Y.lipolytica *Wratislavia K1 was used, a lower concentration of citric acid (about 87-89 g/L) and a high concentration of erythritol (up to 47 g/L) were obtained [39]. It was in line with the results shown by Rymowicz *et al*. [40]. Further, *Y. lipolytica *Wratislavia K1 proved to be superior to other strains by producing erythritol and not citric acid from crude glycerol under optimal conversion conditions, which may be a valuable development [41]. *Y.lipolytica *LGAM S (7)1 also showed potential for converting crude glycerol to citric acid [42]. More recently, it was reported that *Y. lipolytica *N15 could produce citric acid in high amounts, specifically, up to 98 g/L of citric acid and 71 g/L of citric acid were produced from pure glycerol medium and crude glycerol medium, respectively [43].

##### Hydrogen and other lower molecule fuels

The bacterium *Rhodopseudomonas palustris *was capable of photofermentative conversion of crude glycerol to hydrogen. Nearly equal productions were obtained from crude glycerol and pure glycerol. Up to 6 moles H_2 _per mole glycerol were obtained, which was 75% of theoretical. Both rates and yields of hydrogen production could be modified by changing the concentration of added nitrogen. However, some technical obstacles, such as enhancing the efficiency of light utilization by the organisms and developing effective photobioreactors, still need to be solved during development of a practical process [44]. When *Enterobacter aerogenes *HU-101 was employed, hydrogen and ethanol were produced at high yields and with high production rates. But the crude glycerol should be diluted with a synthetic medium in order to increase the rate of glycerol utilization [45]. For maximizing hydrogen production, Jitrwung and Yargeau [46] optimized some media compositions of *E. aerogenes *ATCC 35029 fermented crude glycerol process. More recently, it was reported that *K.pneumoniae mutant *strain and nonpathogenic *Kluyvera cryocrescens *S26 were promising for producing ethanol from crude glycerol [47,48]. In addition, crude glycerol, as a co-substrate, could be used to enhance hydrogen and especially methane production during the anaerobic treatment of different feedstocks including the organic fraction of municipal solid wastes, sewage sludge and slaughterhouse wastes [49-51].

##### Poly (hydroxyalkanoates)

Poly(hydroxyalkanoates) (PHA) represent a complex class of naturally occurring bacterial polyesters and have been recognized as good substitutes for non-biodegradable petrochemically produced polymers. Ashby *et al*. [52] reported that crude glycerol could be used to produce PHA polymer. PHB is the most-studied example of biodegradable polyesters belonging to the group of PHA. The study of the feasibility of using crude glycerol for PHB production, with *Paracoccus denitrificans *and *Cupriavidus necator *JMP 134, showed that the resulting polymers were very similar to those obtained from glucose. But the PHB production decreased significantly when NaCl-contaminated crude glycerol was used. The authors suggested that the harmful effect of the NaCl-contaminant could be reduced by mixing crude glycerol from different manufacturers [53]. Further, a process based on the *Cupriavidus necator *DSM 545 fermentation of crude glycerol was designed for the large-scale production of PHB. However, sodium still hindered the cell growth [54]. *Zobellella denitrificans *MW1 could utilize crude glycerol for growth and PHB production to high concentration, especially in the presence of NaCl. Therefore, it was recommended as an attractive option for large-scale production of PHB with crude glycerol [55].

Additionally, when mixed microbial consortia (MMC) was used for PHA production from crude glycerol, it was found that methanol in the crude glycerol was transformed to PHB by MMC. Further, it was estimated that a 10 million gallon per year biodiesel plant would have the potential of producing 20.9 ton PHB [56]. More recent report showed that *Pseudomonas oleovorans *NRRL B-14682 could also be used for PHB production from crude glycerol [57].

##### Docosahexaenoic acid

A series of papers on the production of docosahexaenoic acid (DHA)-rich algae were published, using crude glycerol, by fermentation of the alga *Schizochytrium limacinum*. For supporting alga growth and DHA production, 75-100 g/L concentration of crude glycerol was recommended as the optimal range. The algal DHA yield was influenced significantly by temperature and ammonium acetate concentration. The optimal amounts for temperature and ammonium acetate were 19.2°C and 1.0 g/L, respectively. The highest DHA yield obtained was 4.91 g/L under the optimized culture conditions [58]. Different sources of crude glycerol did not result in significant variations in algal biomass compositions. The resulting algae had a similar content of DHA and a comparable nutritional profile to commercial algal biomass. That proposed good potential for using crude glycerol-derived algae in omega-3-fortified foods or feeds [59]. Further, DHA-containing algae have been developed as replacements for fish oil for omega-3 fatty acids [60]. Crude glycerol was used to produce fungal biomass that served as eicosapentaenoic acid (EPA)-fortified foods or feeds through fungal fermentation with fungus *Pythium irregulare*. Growing in medium containing 30 g/L crude glycerol and 1.0 g/L yeast extract, the EPA yield and productivity could reach 90 mg/L and 14.9 mg/L per day, respectively. The resulting EPA content was low compared to microalgae for EPA. Optimizing culture conditions and developing high cell density culture techniques are imperative in future work [60]. Recently, it was reported that continuous culture was an effective approach for studying the growth kinetics and behaviors of the algae on crude glycerol [61].

##### Lipids

As the sole carbon source, crude glycerol could be used to produce lipids which might be a sustainable biodiesel feedstock. For example, crude glycerol could be used for culturing *Schizochytrium limacinum *SR21 and *Cryptococcus curvatus*. *S. limacinum *algal growth and lipid production were affected by the concentrations of glycerol. Higher concentrations of glycerol had negative effects on cell growth. For batch culturing of crude glycerol derived from yellow grease, the optimal glycerol concentrations for untreated and treated crude glycerol were 25 and 35 g/L, respectively. With 35 g/L, the obtained highest cellular lipid content was 73.3%. Methanol remaining in crude glycerol could harm *S. limacinum *SR21 growth [62]. For *C. curvatus *yeast, fed-batch was a better process than batch for lipid production. Culturing for 12 days, the lipid content from one-stage fed-batch operation and two-stage fed-batch process were 44.2% and 52%, respectively. Methanol did not have significant inhibitory effect on cell growth. The produced lipid had high concentration of monounsaturated fatty acid and was good biodiesel feedstock [63].

Further, Saenge *et al*. [64] presented that oleaginous red yeast *Rhodotorula glutinis *TISTR 5159, cultured on crude glycerol, produced lipids and carotenoids. The addition of ammonium sulfate and Tween 20 increased the accumulation of lipids and carotenoids. When fed-batch fermentation was employed, the highest lipid content, lipid yield and carotenoids production were 10.05 g/L, 60.7% and 6.10 g/L, respectively. *Chlorella protothecoides *also converted crude glycerol to lipids. The lipids yield was 0.31 g lipids/g substrate [65]. Similarly, with *C. protothecoides *and crude glycerol (62% purity), Chen and Walker [66] demonstrated that the maximum lipid productivity of 3 g/L per day was obtained in a fed-batch operation, which was higher than that produced by batch process. Additionally, Chatzifragkou *et al*. [67] studied the potential of fifteen eukaryotic microorganisms to convert crude glycerol to metabolic products. The results showed that yeast accumulated limited lipids (up to 22 wt.%, wt/wt, in the case of *Rhodotorula sp*.), while fungi accumulated higher amounts of lipids in their mycelia (ranging between 18.1 and 42.6%, wt/wt, of dry biomass).

##### Other chemicals

Beyond the chemicals mentioned above, several other processes for producing useful chemicals from crude glycerol via biotransformations have been developed. A continuous cultivation process and a recently isolated bacterium *Basfia succiniciproducens *DD1 were identified for succinic acid production. The process was characterized as having great process stability, attractive production cost, and impossible pathogenicity of the production strain, but the final production strain needs to be examined further for commercial succinic acid production [68]. Via simulation method, Vlysidis *et al*. [69] showed that the succinic acid co-production from crude glycerol, for a 20 years biodiesel plant, would improve the profit of the overall biorefinery by 60%.

Further, crude glycerol, as the sole carbon source, had the potential of producing phytase in industrial scale in high cell density fermentations with recombinant *Pichia pastoris *possessing a pGAP-based constitutive expression vector [70] and producing butanol with *Clostridium pasteurianum*. The highest yield of butanol was 0.30 g/g, which was significantly higher than the 0.15-0.20 g/g butanol yield typically obtained during the fermentation of glucose using *Clostridium acetobutylicum*. However, further understanding and optimizing of the process are still needed. It remained unclear what impact the impurities in crude glycerol would have on the solvent formation [71]. Similarly, crude glycerol could be used in a bioprocess with *P. pastoris *without any purification. Canola oil-derived crude glycerol was the most favorable carbon source and showed great potential for the production of additional value-added products such as the recombinant human erythropoietin and cell growth [72]. Crude glycerol also could be economic carbon and nutrient sources for bacterial cellulose (BC) production. The BC amount obtained was about 0.1 g/L after 96 h incubation. The addition of other nutrient sources (yeast extract, nitrogen and phosphate) to crude glycerol culture media increased the BC production by ~200% [73].

Additionally, *Gluconobacter *sp. NBRC3259 could be used to produce glyceric acid from crude glycerol with an activated charcoal pretreatment. 49.5 g/L of glyceric acid and 28.2 g/L dihydroxyacetone were produced from 174 g/L of glycerol [74]. When *Staphylococcus caseolyticus *EX17 was employed, crude glycerol could be used for solvent tolerant lipase production [75]. More recently, it was reported that *Ustilago maydis *was a good biocatalyst for converting crude glycerol to glycolipid-type biosurfactants and other useful products [76]. Fungal protein, *Rhizopus microsporus var. oligosporus*, production on crude glycerol was another potential use of crude glycerol. The obtained fungal biomass contained high amounts of threonine and could be co-fed with commercial sources. But feeding formulation need be further studied [77].

#### Chemicals produced through conventional catalytic conversions

##### Oxygenated chemicals

As fuel additive, the oxygenate synthesized compound, (2,2-dimethyl-1,3-dioxolan-4-yl) methyl acetate, could be produced from crude glycerol and used as a biodiesel additive. It could improve biodiesel viscosity and could meet the requirements established for diesel and biodiesel fuels by the American and European Standards (ASTM D6751 and EN 14214, respectively) for flash point and oxidation stability. This new compound could compete with other biodiesel additives [78]. Further, acrolein is an important starting chemical for producing detergents, acrylic acid ester and super absorber polymers. Sereshki *et al*. [79] reported a process involving adding liquid crude glycerol directly into a fluidized bed reactor, vaporizing it, and then reacting them to produce acrolein over a tungsten doped zirconia catalyst. In this process, glycerol evaporated in the fluidized bed reactor leaving behind salt crystals which were only loosely bound to the surface and could be separated from the catalyst using mechanical agitation. This process has the potential to reduce the accumulation of salt in the reactor.

##### Hydrogen or syngas

Crude glycerol was proven to be a viable alternative for producing hydrogen or syngas [80,81]. Gasification was the main employed technique. Thermo-gravimetry coupled with FTIR spectroscopy analysis proved that the thermal decomposition mechanism of crude glycerol mainly involved four phases and CO_2_, H_2_, CH_4_, and CO were the main gas products [82]. Gasification with in situ CO_2 _removal was effective and had high energy efficiency [83]. Supercritical water gasification of crude glycerol was performed under uncatalyzed and alkaline catalyzed conditions. The reaction temperature determined the decomposition degree. When NaOH was employed as catalyst nearly 90 vol.% of the product gas was H_2 _and no char was produced [84]. Additionally, co-gasification of crude glycerol and hardwood chips, in a downdraft gasifier, was another promising option for utilizing crude glycerol. The loading amount of crude glycerol had significant influence on CO and CH_4 _concentration, while having no effect on H_2 _and CO_2 _yield. The study suggested that the co-gasification could perform well in downdraft gasifiers with hardwood chips mixing with liquid crude glycerol up to 20 wt.% [85].

##### Other chemicals from conventional catalytic conversions

Addition to the previous mentioned chemicals produced from crude glycerol, several other chemicals from conventional catalytic conversion processes were reported. Monoglycerides could be produced via glycerolysis of triglyceride with crude glycerol. A two-step process, involving the removal of residual glycerol and crystallization, was employed for purification of the monoglycerides produced from glycerolysis. An approximate 99% purity monopalmitin was achieved [86]. More recently, the glycerolysis of soybean oil, using crude glycerol, was investigated. The amount of inorganic alkaline catalyst in the crude glycerol affected the concentration of the produced monoglycerides. Crude glycerol containing lower content of inorganic catalyst (about 2.7 wt.% NaOH) produced the highest concentration of monoglycerides, which was about 42% [87].

### Other uses

Beyond the aforementioned uses, a few other potential applications of crude glycerol have been reported. Crude glycerol was used as a high-boiling-point organic solvent to enhance enzymatic hydrolysis of lignocellulosic biomass during atmospheric autocatalytic organosolv pretreatment [88]. Crude glycerol (without any purification) also could be used as a green solvent for organic reactions. Two representative reactions were base catalyzed aldol condensation and palladium catalyzed Heck carbon-carbon coupling [89]. Functioning as an organic carbon source for the removal of nitrate in the wastewater denitrification process, the denitrification efficiency could be increased by 2.0-5.0 mg NO_3_-N L^-1 ^per crude glycerol dose of 100 L [90]. Additionally, crude glycerol could be used as a fuel for generating electricity from microbial fuel cells [91]. Both co-hydrothermal pyrolysis and co-liquefaction of manure with crude glycerol could improve the production yield of bio-oil. However, for the co-liquefaction, too much addition of crude glycerol affected the carbon content and heat value of the bio-oil [92,93].

## Conclusions

Effective utilization of crude glycerol is very crucial to the commercialization and further development of biodiesel production. In the long term, utilization of biomass-derived glycerol will not only contribute to reducing society's dependence on nonrenewable resources but also will promote the development of integrated biorefineries.

This review addresses the value-added opportunities for crude glycerol from biodiesel production, mainly as a feedstuff for animal feed and as feedstocks for chemicals. Promising results have been achieved by researchers, especially for non-ruminant animals such as pigs, laying hens, and broilers. However, there still are some considerations that need to be taken into account for broad-scale use of this biomass-derived chemical in animal feeds. First of all, the chemical composition of crude glycerol varies significantly with the methods and feedstocks used to produce biodiesel. Since different researchers have used different qualities of crude glycerol for their studies, animal producers need to pay attention when they decide to include crude glycerol as a component of animal feed rations. Secondly, to some extent the impurities in crude glycerol affect feed performance. Finally, the amount of crude glycerol included in feed formulations needs to be considered. The establishment of a crude glycerol feed specification is recommended. The resulting "standard" crude glycerol would have a higher value simply because it would be consistent from all producers.

Conventional catalysis and biotransformation are the two main routes available for converting crude glycerol to a variety of value-added chemicals. In more recent years, there have been extensive studies, and some encouraging results, on processes for crude glycerol conversion. For example, the productions of 1,3-propanediol, citric acid, poly (hydroxyalkanoates), butanol, hydrogen, docosahexaenoic acid-rich algae, monoglycerides, lipids and syngas from crude glycerol are promising. However, many of the technologies offered still need further development to make them cost-effective and operationally feasible for incorporation into biorefineries.

Impurities in crude glycerol can greatly influence the conversion of glycerol into other products. In some biological conversion processes, pollutants in crude glycerol, inhibit cell and fungal growth and result in lower production rates and product yields (compared with pure or commercial glycerol under the same culture conditions). On the other hand, for conventional catalytic conversions, impurities poison the catalysts, increasing char production and influencing product yield. Many of the technologies need to be more fully understood and optimized, such as optimizing reaction parameters, production yields, and fermentation conditions, developing mutant strains and efficient bioreactors for stable cultures, and improving the activity and selectivity of catalysts. Additionally, cell growth has been shown to be inhibited during fermentation when the initial crude glycerol concentration was high. Conversely, higher crude glycerol concentrations are necessary for improving production efficiency.

In brief, it has been shown that biological conversions of crude glycerol can produce higher value chemicals. However, it is impossible for the current state of the technologies to convert glycerol at rates necessary to prevent a large accumulation of glycerol. Both improved biological and conventional techniques will be needed in future biodiesel plants. Other promising uses, starting with crude glycerol, also have been disclosed. Although hopeful results have been achieved, there are still aspects that need to be improved.

In summary, in recent years, scores of utilization opportunities of crude glycerol have been presented and promising results have been achieved. However, it is imperative to point out that there are still more technical hurdles to jump for developing practical processes to directly utilize crude glycerol from biodiesel production on a large scale. Quick overviews of what has been investigated, with respect to potential chemicals from biological and conventional catalytic conversions of crude glycerol, are summarized in Tables 1 and 2, respectively.

**Table 1:** Biological conversions of crude glycerol to chemicals

Product	Pathway	Product productivity	Reference
1, 3-propanediol	Fed-batch cultures of *Klebsiella pneumoniae *strain	1.7 g/L/h	[6]
	Maximum 1,3-propanediol production from *K. pneumonia*	13.8 g/L	[29]
	Optimize 1,3-propanediol production from *K. pneumoniae *ATCC 15380	56 g/L	[30]
	Integrated bioprocess combining biodiesel production by lipase with microbial production of 1, 3-propanediol by *K. pneumoniae *strain	1.7 g/L/h	[31]
	*Clostridium butyricum *strain VPI 3266 on a synthetic medium	0.60 mol/mol glycerol	[32]
	*C. butyricum *strain F2b (process modelling)	NA^a^	[33]
	*C. butyricum *VPI 1718; fed-batch operation under non-sterile culture conditions	67.9 g/L	[35]
	One vessel bio- and chemocatalytic process; in a biphasic system without intermediate separation of 1, 3-propanediol; *C. butyricum *DSM10703	134 m mol/L	[36]
Citric acid	*Y. lipolytica *strain ACA-DC 50109 (process modelling)	NA^a^	[33]
	Acetate Mutants of *Y. lipolytica *Wratislavia AWG7 strain; Fed-batch operation	139 g/L	[39]
	*Yarrowia lipolytica *strain LGAM S (7)1	35 g/L	[42]
	*Y. lipolytica *N15	71 g/L	[43]
Erythritol	Fed-batch cultures of *Y. lipolytica *Wratislavia K1	1 g/L/h	[41]
Hydrogen	Photofermentative conversion process; *Rhodopseudomonas palustris *strain	6 mol/mol glycerol	[44]
	*Enterobacter aerogenes *strain HU-101; continuous culture; porous ceramics as a support material to fix cells	63 mmol/L/h	[45]
	Optimize some media compositions of *E. aerogenes *ATCC 35029	0.85 mol/mol glycerol	[46]
	Anaerobic treatment process; crude glycerol was a co-substrate	2.9 mmol/g glycerol	[49]
Poly (hydroxyalkanoates) (PHAs)	*Pseudomonas oleovorans *NRRL B-14682 and *P. corrugata *388 grew and synthesized PHB and mcl-PHA, respectively	NA^a^	[52]
	Producing PHB; *Paracoccus denitrificans *and *Cupriavidus necator *JMP 134 strains	48%	[53]
	Producing PHB; *Cupriavidus necator *strain DSM 545	50%	[54]
	Producing PHB; *Zobellella denitrificans *MW1; fed-batch cultivation	66.9% ± 7.6%	[55]
	Producing PHB; Mixed microbial consortia (MMC)	> 50%	[56]
	*Pseudomonas oleovorans *NRRL B-14682; batch culture	30%	[57]
Phytase	High cell density fermentations, recombinant *Pichia pastoris*	1125 U/mL	[70]
Lipase	*Staphylococcus caseolyticus *EX17	127.3 U/L	[75]
Succinic acid	*Basfia succiniciproducens *DD1; continuous cultivation process	1.02 g/g glycerol	[68]
Docosahexaenoic acid-rich algae	Fermentation of the alga, *Schizochytrium limacinum *strain; continuous culture	0.52 g/L-day	[61]
Eicosapentaenoic acid	Fungus *Pythium irregulare*	90 mg/L	[60]
Lipid	*Schizochytrium limacinum *SR21; batch culture	73.3%	[62]
	*Cryptococcus curvatus*; two-stage fed-batch process	52%	[63]
	Oleaginous red yeast *Rhodotorula glutinis *TISTR 5159; fed-batch fermentation	60.7%	[65]
	Chlorella protothecoides	3 g/L per day	[66]
	Fungi	42.6%	[67]
Recombinant human erythropoietin etc.	*Pichia pastoris *medium	31 mg/L	[72]
Ethanol	Nonpathogenic *Kluyvera cryocrescens *S26; batch fermentation	27 g/L	[47]
	*Klebsiella pneumoniae *mutant strain (GEM167)	21.5 g/L	[48]
Methane	Anaerobic digestion	0.306 m^3^/kg glycerol	[51]
Butanol	*Clostridium pasteurianum *(ATCC^®^6013™)	0.30 g/g glycerol	[71]
Fungal protein	*Rhizopus microsporus var. oligosporus*	0.83 ± 0.02 g/g glycerol	[77]

^a ^represents data are not available

**Table 2:** Conventional catalytic conversions of crude glycerol to chemicals

Product	Pathway	Product productivity	Reference
(2,2-dimethyl-1,3-dioxolan-4-yl) methyl acetate	Oxygenate synthesized compound	NA^a^	[78]
Acrolein	Fuidized bed, tungsten doped zirconia catalyst	21%	[79]
Monoglyceride	Two-step process, purification of the monoglyceride produced from glycerolysis of palm stearin	~99% purity	[86]
	Glycerolysis of soybean oil	~42%	[87]
Gaseous products	Steam reforming process; platinum alumina as catalyst	NA^a^	[80]
	Thermal decomposition of crude glycerol by pyrolysis	NA^a^	[82]
	Steam gasification with in situ CO_2 _removal	88 vol.% H_2 _purity	[83]
	Hydrothermal reforming of crude glycerol	~90 vol.% H_2 _purity	[84]
	Co-gasification of crude glycerol and hardwood chips	NA^a^	[85]

^a ^represents data are not available

## Abbreviations

DE: Digestible energy; ME: Metabolizable energy; AMEn: nitrogen-corrected apparent ME; GE: General energy; PHA: Poly(hydroxyalkanoates); MMC: Mixed microbial consortia; DHA: Docosahexaenoic acid; EPA: Eicosapentaenoic acid; BC: Bacterial cellulose.

## Competing interests

The authors declare that they have no competing interests.

## Authors' contributions

MAH conceived the study and participated in the design of this review manuscript, and revised the manuscript critically for important intellectual content and language. RS participated in the coordination and helped draft the manuscript. FY carried out the collecting of the references and the drafting of the manuscript. All authors read and approved the final manuscript.

## Authors' information

MAH, Professor Emeritus of Biological System Engineering and Director of the Industrial Agricultural Products Center at the University of Nebraska, Lincoln. Research interests are mainly in the area of value-added process engineering; uses of vegetable oils and animal fats in biofuel and lubricant applications; extrusion processes for production of levulinic acid, microcrystalline cellulose and modified starches; use of plant residues and other waste streams for biopower generation.

RCS, Director of Institute of Biomass Chemistry and Technology in Beijing Forestry University, Beijing, China. Research interests focus mainly on fundamental research of biomass from farming and forestry wastes on valuable application.

FXY, PhD, research interests focus mainly on biofuels and biomass utilizations. She investigated the production of biodiesel and the utilization of the crude glycerol from biodiesel production during her graduate study.

## References

[B1] Biodiesel 2020 Global Market Survey, Feedstock Trends and Forecasts Emerging Markets Online 2008 2 http://www.healthtech.com/biodiesel2020 Multi-Client Study

[B2] Anand P Saxena RK A comparative study of solvent-assisted pretreatment of biodiesel derived crude glycerol on growth and 1,3-propanediol production from *Citrobacter freundi* New Biotechnol 2011 00 1 7 10.1016/j.nbt.2011.05.010 21689798

[B3] Kerr BJ Dozier WA III Bregendahl K Nutritional value of crude glycerin for nonruminants Proceedings of the 23rd Annual Carolina Swine Nutrition Conference 2007 Raleigh, NC 6 18

[B4] Hansen CF Hernandez A Mullan BP Moore K Trezona-Murray M King RH Pluske JR A chemical analysis of samples of crude glycerol from the production of biodiesel in Australia, and the effects of feeding crude glycerol to growing-finishing pigs on performance, plasma metabolites and meat quality at slaughter Anim Prod Sci 2009 49 154 161 10.1071/EA08210

[B5] Asad-ur-Rehman Saman WRG Nomura N Sato S Matsumura M Pre-treatment and utilization of raw glycerol from sunflower oil biodiesel for growth and 1, 3-propanediol production by *Clostridium butyricum* J Chem Technol Biotechnol 2008 83 1072 1080

[B6] Mu Y Teng H Zhang DJ Wang W Xiu ZL Microbial production of 1, 3-propanediol by *Klebsiella pneumonia *using crude glycerol from biodiesel preparations Biotechnol Lett 2006 28 1755 1759 10.1007/s10529-006-9154-z 16900328

[B7] Lancrenon X Fedders J An innovation in glycerin purification Biodiesel Magazine http://www.biodieselmagazine.com/articles/2388/an-innovation-in-glycerin-purification

[B8] Pagliaro M Rossi M The future of glycerol: new uses of a versatile raw material R Soc Chem (Great Britain) 2008 117

[B9] Nilles D Combating the Glycerin Glut 2006 http://www.biodieselmagazine.com/article.jsp?article_id=1123&q=&page=2

[B10] Fisher LJ Erfle JD Lodge GA Sauer FD Effects of propylene glycol or glycerol supplementation of the diet of dairy cows on feed intake, milk yield and composition, and incidence of ketosis Can J Anim Sci 1973 53 289 296 10.4141/cjas73-045

[B11] Dasari M Crude glycerol potential described Feedstuffs 2007 79 1 3

[B12] Lammers PJ Kerr BJ Weber TE Dozier WA III Kidd MT Bregendahl K Honeyman MS Digestible and metabolizable energy of crude glycerol for growing pigs J Anim Sci 2008 86 602 608 18073284 10.2527/jas.2007-0453

[B13] Lammers PJ Kerr BJ Honeyman MS Stalder K Dozier WA III Weber TE Kidd MT Bregendahl K Nitrogen-corrected apparent metabolizable energy value of crude glycerol for laying hens Poult Sci 2008 87 104 107 10.3382/ps.2007-00255 18079458

[B14] Dozier WA III Kerr BJ Corzo A Kidd MT Weber TE Bregendahl K Apparent metabolizable energy of glycerin for broiler chickens Poult Sci 2008 87 317 322 10.3382/ps.2007-00309 18212375

[B15] Kerr BJ Weber TE Dozier WA III Kidd MT Digestible and metabolizable energy content of crude glycerin originating from different sources in nursery pigs J Anim Sci 2009 87 4042 4049 10.2527/jas.2008-1676 19717766

[B16] Kijora C Kupsch RD Evaluation of technical glycerols from Biodiesel production as a feed component in fattening of pigs Fett/Lipid 1996 98 240 245 10.1002/lipi.19960980703

[B17] Schieck SJ Shurson GC Kerr BJ Johnston LJ Evaluation of glycerol, a biodiesel coproduct, in grow-finish pig diets to support growth and pork quality J Anim Sci 2010 88 3927 3935 10.2527/jas.2010-2858 20656974

[B18] Shields MC Heugten EV Lin X Odle J Stark CS Evaluation of the nutritional value of glycerol for nursery pigs J Anim Sci 2011 89 2145 2153 10.2527/jas.2010-3558 21297059

[B19] Schieck SJ Kerr BJ Baidoo SK Shurson GC Johnston LJ Use of crude glycerol, a biodiesel coproduct, in diets for lactating sows J Anim Sci 2010 88 2648 2656 10.2527/jas.2009-2609 20382881

[B20] Swiatkiewicz S Koreleski J Effect of crude glycerin level in the diet of laying hens on egg performance and nutrient utilization Poult Sci 2009 88 615 619 10.3382/ps.2008-00303 19211533

[B21] McLea L Ball MEE Kilpatrick D Elliott C The effect of glycerol inclusion on broiler performance and nutrient digestibility Br Poult Sci 2011 52 368 375 10.1080/00071668.2011.584520 21732883

[B22] Cerrate S Yan F Wang Z Coto C Sacakli P Waldroup PW Evaluation of glycerine from biodiesel production as a feed ingredient for broilers Int J Poult Sci 2006 5 1001 1007

[B23] Gunn PJ Neary MK Lemenager RP Lake SL Effects of crude glycerin on performance and carcass characteristics of finishing wether lambs J Anim Sci 2010 88 1771 1776 10.2527/jas.2009-2325 20154165

[B24] Hampy KR Kellogg DW Coffey KP Kegley EB Caldwell JD Lee MS Akins MS Reynolds JL Moore JC Southern KD Glycerol as a Supplemental Energy Source for Meat Goats AAES Research Series 2008 553 63 64

[B25] Donkin SS Glycerol from biodiesel production: the new corn for dairy cattle Rev Bras Zootec 2008 37 280 286 10.1590/S1516-35982008001300032

[B26] Donkin SS Koser SL White HM Doane PH Cecava MJ Feeding value of glycerol as a replacement for corn grain in rations fed to lactating dairy cows J Dairy Sci 2009 92 5111 5119 10.3168/jds.2009-2201 19762829

[B27] Parsons GL Shelor MK Drouillard JS Performance and carcass traits of finishing heifers fed crude glycerin J Anim Sci 2009 87 653 657 18849392 10.2527/jas.2008-1053

[B28] Cecava M Doane P Holzgraefe D Application of Crude Glycerin for Improved Livestock Production US Patent Appl 2008/0260896 A1 published 10/23/2008

[B29] Oh BR Seo JW Choi MH Kim CH Optimization of culture conditions for 1, 3-propanediol production from crude glycerol by *Klebsiella pneumonia *using response surface methodology Biotechnol Bioprocess Eng 2008 13 666 670 10.1007/s12257-008-0090-8

[B30] Hiremath A Kannabiran M Rangaswamy V 1, 3-Propanediol production from crude glycerol from jatropha biodiesel process New Biotechnol 2011 28 19 23 10.1016/j.nbt.2010.06.006 20601262

[B31] Mu Y Xiu ZL Zhang DJ A combined bioprocess of biodiesel production by lipase with microbial production of 1, 3-propanediol by *Klebsiella pneumonia* Biochem Eng J 2008 40 537 541 10.1016/j.bej.2008.02.011

[B32] González-Pajuelo M Andrade JC Vasconcelos I Production of 1, 3-propanediol by *Clostridium butyricu *VPI 3266 using a synthetic medium and raw glycerol J Ind Microbiol Biotechnol 2004 31 442 446 10.1007/s10295-004-0168-z 15378388

[B33] Papanikolaou S Aggelis G Modelling aspects of the biotechnological valorization of raw glycerol: production of citric acid by *Yarrowia lipolytic *and 1, 3-propanediol by *Clostridium butyricu* J Chem Technol Biotechnol 2003 78 542 547 10.1002/jctb.831

[B34] Papanikolaou S Fakas S Fick M Chevalot I Galiotou-Panayotou M Komaitis M Marc I Aggelis G Biotechnological valorisation of raw glycerol discharged after bio-diesel (fatty acid methyl esters) manufacturing process: Production of 1, 3-propanediol, citric acid and single cell oil Biomass Bioenergy 2008 32 60 71 10.1016/j.biombioe.2007.06.007

[B35] Chatzifragkou A Papanikolaou S Dietz D Doulgeraki AI Nychas GJE Zeng AP Production of 1, 3-propanediol by *Clostridium butyricu *growing on biodiesel-derived crude glycerol through a non-sterilized fermentation process Appl Microbiol Biotechnol 2011 91 101 112 10.1007/s00253-011-3247-x 21484206

[B36] Liu S Rebros M Stephens G Marr AC Adding value to renewables: a one pot process combining microbial cells and hydrogen transfer catalysis to utilise waste glycerol from biodiesel production Chem Commun 2009 17 2308 2310 10.1039/b820657k 19377668

[B37] Chatzifragkou A Dietz D Komaitis M Zeng AP Papanikolaou S Effect of Biodiesel-Derived Waste Glycerol Impurities on Biomass and 1,3-Propanediol Production of *Clostridium butyricu *VPI 1718 Biotechnol Bioeng 2010 107 76 84 10.1002/bit.22767 20506102

[B38] Papanikolaou S Aggelis G Biotechnological valorization of biodiesel derived glycerol waste through production of single cell oil and citric acid by *Yarrowia lipolytic* Lipid Technol 2009 21 83 87 10.1002/lite.200900017

[B39] Rywińska A Rymowicz W Żlarowska B Biosynthesis of Citric Acid from Glycerol by Acetate Mutants of *Yarrowia lipolytic *in Fed-Batch Fermentation Food Technol Biotechnol 2009 47 1 6

[B40] Rymowicz W Rywińska A Gladkowski W Simultaneous production of citric acid and erythritol from crude glycerol by *Yarrowia lipolytic *Wratislavia K1 Chem Pap 2008 62 239 246 10.2478/s11696-008-0018-y

[B41] Rymowicz W Rywińska A Marcinkiewicz M High-yield production of erythritol from raw glycerol in fed-batch cultures of *Yarrowia lipolytic* Biotechnol Lett 2009 31 377 380 10.1007/s10529-008-9884-1 19037599

[B42] Papanikolaou S Muniglia L Chevalot I Aggelis G Marc I *Yarrowia lipolytic *as a potential producer of citric acid from raw glycerol J Appl Microbiol 2002 92 737 744 10.1046/j.1365-2672.2002.01577.x 11966915

[B43] Kamzolova SV Fatykhova AR Dedyukhina EG Anastassiadis SG Golovchenko NP Morgunov IG Citric Acid Production by Yeast Grown on Glycerol-Containing Waste from Biodiesel Industry Food Technol Biotechnol 2011 49 65 74

[B44] Sabourin-Provost G Hallenbeck PC High yield conversion of a crude glycerol fraction from biodiesel production to hydrogen by photofermentation Bioresour Technol 2009 100 3513 3517 10.1016/j.biortech.2009.03.027 19339176

[B45] Ito T Nakashimada Y Senba K Matsui T Nishio N Hydrogen and ethanol production from glycerol-containing wastes discharged after biodiesel manufacturing process J Biosci Bioeng 2005 100 260 265 10.1263/jbb.100.260 16243274

[B46] Jitrwung R Yargeau V Optimization of media composition for the production of biohydrogen from waste glycerol Int J Hydrogen Energy 2011 36 9602 9611 10.1016/j.ijhydene.2011.05.092

[B47] Choi WJ Hartono MR Chan WH Yeo SS Ethanol production from biodiesel-derived crude glycerol by newly isolated *Kluyvera cryocrescen* Appl Microbiol Biotechnol 2011 89 1255 1264 10.1007/s00253-010-3076-3 21212944

[B48] Oh BR Seo JW Heo SY Hong WK Luo LH Joe M Park DH Kim CH Efficient production of ethanol from crude glycerol by a *Klebsiella pneumonia *mutant strain Bioresour Technol 2011 102 3918 3922 10.1016/j.biortech.2010.12.007 21186120

[B49] Fountoulakis MS Manios T Enhanced methane and hydrogen production from municipal solid waste and agro-industrial by-products co-digested with crude glycerol Bioresour Technol 2009 100 3043 3047 10.1016/j.biortech.2009.01.016 19231165

[B50] Fountoulakis MS Petousi I Manios T Co-digestion of sewage sludge with glycerol to boost biogas production Waste Manage 2010 30 1849 1853 10.1016/j.wasman.2010.04.011 20434322

[B51] López JÁS Santos MDM Pérez AFC Martín AM Anaerobic digestion of glycerol derived from biodiesel manufacturing Bioresour Technol 2009 100 5609 5615 10.1016/j.biortech.2009.06.017 19592236

[B52] Ashby RD Solaiman DKY Foglia TA Bacterial poly (hydroxyalkanoate) polymer production from the biodiesel co-product stream J Polym Environ 2004 12 105 112

[B53] Mothes G Schnorpfeil C Ackermann JU Production of PHB from crude glycerol Eng Life Sci 2007 7 475 479 10.1002/elsc.200620210

[B54] Cavalheiro JMBT De Almeida M Grandfils C Da Fonseca M Poly (3-hydroxybutyrate) production by *Cupriavidus necato *using waste glycerol Process Biochem 2009 44 509 515 10.1016/j.procbio.2009.01.008

[B55] Ibrahim MHA Steinbüchel A Poly (3-Hydroxybutyrate) Production from Glycerol by *Zobellella denitrifican *MW1 via High-Cell-Density Fed-Batch Fermentation and Simplified Solvent Extraction Appl Environ Microbiol 2009 75 6222 6231 10.1128/AEM.01162-09 19666728 PMC2753068

[B56] Dobroth ZT Hu S Coats ER McDonald AG Polyhydroxybutyrate synthesis on biodiesel wastewater using mixed microbial consortia Bioresour Technol 2011 102 3352 3359 10.1016/j.biortech.2010.11.053 21130645

[B57] Ashby RD Solaiman DKY Strahan GD Efficient Utilization of Crude Glycerol as Fermentation Substrate in the Synthesis of Poly(3-hydroxybutyrate) Biopolymers J Am Oil Chem Soc 2011 88 949 959 10.1007/s11746-011-1755-6

[B58] Chi Z Pyle D Wen Z Frear C Chen S A laboratory study of producing docosahexaenoic acid from biodiesel-waste glycerol by microalgal fermentation Process Biochem 2007 42 1537 1545 10.1016/j.procbio.2007.08.008

[B59] Pyle DJ Garcia RA Wen Z Producing docosahexaenoic acid (DHA)-rich algae from biodiesel-derived crude glycerol: effects of impurities on DHA production and algal biomass composition J Agric Food Chem 2008 56 3933 3939 10.1021/jf800602s 18465872

[B60] Athalye SK Garcia RA Wen ZY Use of Biodiesel-Derived Crude Glycerol for Producing Eicosapentaenoic Acid (EPA) by the Fungus *Pythium irregular* J Agric Food Chem 2009 57 2739 2744 10.1021/jf803922w 19265450

[B61] Ethier S Woisard K Vaughan D Wen ZY Continuous culture of the microalgae *Schizochytrium limacinu *on biodiesel-derived crude glycerol for producing docosahexaenoic acid Bioresour Technol 2011 102 88 93 10.1016/j.biortech.2010.05.021 20570140

[B62] Liang YN Sarkany N Cui Y Blackburn JW Batch stage study of lipid production from crude glycerol derived from yellow grease or animal fats through microalgal fermentation Bioresour Technol 2010 101 6745 6750 10.1016/j.biortech.2010.03.087 20381345

[B63] Liang YN Cui Y Trushenski J Blackburn JW Converting crude glycerol derived from yellow grease to lipids through yeast fermentation Bioresour Technol 2010 101 7581 7586 10.1016/j.biortech.2010.04.061 20478702

[B64] Saenge C Cheirsilp B Suksaroge TT Bourtoom T Potential use of oleaginous red yeast *Rhodotorula glutini *for the bioconversion of crude glycerol from biodiesel plant to lipids and carotenoids Process Biochem 2011 46 210 218 10.1016/j.procbio.2010.08.009

[B65] O'Grady J Morgan JA Heterotrophic growth and lipid production of *Chlorella protothecoide *on glycerol Bioprocess Biosyst Eng 2011 34 121 125 10.1007/s00449-010-0474-y 20976474

[B66] Chen YH Walker TH Biomass and lipid production of heterotrophic microalgae *Chlorella protothecoide *by using biodiesel-derived crude glycerol Biotechnol Lett 2011 33 1973 1983 10.1007/s10529-011-0672-y 21691839

[B67] Chatzifragkou A Makri A Belka A Bellou S Mavrou M Mastoridou M Mystrioti P Onjaro G Aggelis G Papanikolaou S Biotechnological conversions of biodiesel derived waste glycerol by yeast and fungal species Energy 2011 36 1097 1108 10.1016/j.energy.2010.11.040

[B68] Scholten E Renz T Thomas J Continuous cultivation approach for fermentative succinic acid production from crude glycerol by *Basfia succiniciproducen *DD1 Biotechnol Let 2009 31 1947 1951 10.1007/s10529-009-0104-4 19705071

[B69] Vlysidis A Binns M Webb C Theodoropoulos C A techno-economic analysis of biodiesel biorefineries: Assessment of integrated designs for the co-production of fuels and chemicals Energy 2011 36 4671 4683 10.1016/j.energy.2011.04.046

[B70] Tang S Boehme L Lam H Zhang Z *Pichia pastori *fermentation for phytase production using crude glycerol from biodiesel production as the sole carbon source Biochem Eng J 2009 43 157 162 10.1016/j.bej.2008.09.020

[B71] Taconi KA Venkataramanan KP Johnson DT Growth and solvent production by *Clostridium pasteurianu *ATCC^® ^6013™ utilizing biodiesel-derived crude glycerol as the sole carbon source Environ Prog Sustainable Energy 2009 28 100 110 10.1002/ep.10350

[B72] Çelik E Ozbay N Oktar N Çalik P Use of biodiesel byproduct crude glycerol as the carbon source for fermentation processes by recombinant *Pichia pastori* Ind Eng Chem Fundam 2008 47 2985 2990

[B73] Carreira P Mendes JAS Trovatti E Serafim LS Freire CSR Silvestre AJD Neto CP Utilization of residues from agro-forest industries in the production of high value bacterial cellulose Bioresour Technol 2011 102 7354 7360 10.1016/j.biortech.2011.04.081 21601445

[B74] Habe H Shimada Y Fukuoka T Kitamoto D Itagaki M Watanabe K Yanagishita H Sakaki K Production of glyceric acid by *Gluconobacte *sp NBRC3259 using raw glycerol Biosci, Biotechnol, Biochem 2009 73 1799 1805 10.1271/bbb.90163 19661679

[B75] Volpato G Rodrigues RC Heck JX Ayub MAZ Production of organic solvent tolerant lipase by *Staphylococcus caseolyticu *EX17 using raw glycerol as substrate J Chem Technol Biotechnol 2008 83 821 828 10.1002/jctb.1875

[B76] Liu Y Koh CMJ Ji L Bioconversion of crude glycerol to glycolipids in *Ustilago maydi* Bioresour Technol 2011 102 3927 3933 10.1016/j.biortech.2010.11.115 21186122

[B77] Nitayavardhana S Khanal SK Biodiesel-derived crude glycerol bioconversion to animal feed: A sustainable option for a biodiesel refinery Bioresour Technol 2011 102 5808 5814 10.1016/j.biortech.2011.02.058 21382713

[B78] García E Laca M Pérez E Garrido A Peinado J New Class of Acetal Derived from Glycerin as a Biodiesel Fuel Component Energy Fuels 2008 22 4274 4280 10.1021/ef800477m

[B79] Sereshki BR Balan SJ Patience GS Dubois JL Reactive Vaporization of Crude Glycerol in a Fluidized Bed Reactor Ind Eng Chem Res 2010 49 1050 1056 10.1021/ie9006968

[B80] Slinn M Kendall K Mallo C Andrews J Steam reforming of biodiesel by-product to make renewable hydrogen Bioresour Technol 2008 99 5851 5858 10.1016/j.biortech.2007.10.003 18032034

[B81] Yoon SJ Choi Y Son Y Lee SH Lee JG Gasification of biodiesel by-product with air or oxygen to make syngas Bioresour Technol 2010 101 1227 1232 10.1016/j.biortech.2009.09.039 19819133

[B82] Dou B Dupont V Williams PT Chen H Ding Y Thermogravimetric kinetics of crude glycerol Bioresour Technol 2009 100 2613 2620 10.1016/j.biortech.2008.11.037 19167215

[B83] Dou B Rickett GL Dupont V Williams PT Chen H Ding Y Steam reforming of crude glycerol with in situ CO_2 _sorption Bioresour Technol 2010 101 2436 2442 10.1016/j.biortech.2009.10.092 19945865

[B84] Onwudili JA Williams PT Hydrothermal reforming of bio-diesel plant waste: Products distribution and characterization Fuel 2010 89 501 509 10.1016/j.fuel.2009.06.033

[B85] Wei L Pordesimo LO Haryanto A Wooten J Co-gasification of hardwood chips and crude glycerol in a pilot scale downdraft gasifier Bioresour Technol 2011 102 6266 6272 10.1016/j.biortech.2011.02.109 21435871

[B86] Chetpattananondh P Tongurai C Synthesis of high purity monoglycerides from crude glycerol and palm stearin Songklanakarin J Sci Technol 2008 30 515 521

[B87] Echeverri DA Cardeno F Rios LA Glycerolysis of Soybean Oil with Crude Glycerol Containing Residual Alkaline Catalysts from Biodiesel Production J Am Oil Chem Soc 2011 88 551 557 10.1007/s11746-010-1688-5

[B88] Sun F Chen H Organosolv pretreatment by crude glycerol from oleochemicals industry for enzymatic hydrolysis of wheat straw Bioresour Technol 2008 99 5474 5479 10.1016/j.biortech.2007.11.001 18077155

[B89] Wolfson A Litvak G Dlugy C Shotland Y Tavor D Employing crude glycerol from biodiesel production as an alternative green reaction medium Ind Crops Prod 2009 30 78 81 10.1016/j.indcrop.2009.01.008

[B90] Bodík I BlŠťáková A Sedláček S Hutnan M Biodiesel waste as source of organic carbon for municipal WWTP denitrification Bioresour Technol 2009 100 2452 2456 10.1016/j.biortech.2008.11.050 19128964

[B91] Feng Y Yang Q Wang X Liu Y Lee H Ren N Treatment of biodiesel production wastes with simultaneous electricity generation using a single-chamber microbial fuel cell Bioresour Technol 2011 102 411 415 10.1016/j.biortech.2010.05.059 20889062

[B92] Xiu S Shahbazi A Shirley V Mims MR Wallace CW Effectiveness and mechanisms of crude glycerol on the biofuel production from swine manure through hydrothermal pyrolysis J Anal Appl Pyrolysis 2010 87 194 198 10.1016/j.jaap.2009.12.002

[B93] Xiu SN Shahbazi A Wallace CW Wang L Cheng D Enhanced bio-oil production from swine manure co-liquefaction with crude glycerol Energy Convers Manage 2011 52 1004 1009 10.1016/j.enconman.2010.08.028

